# The College of Nursing *(Limited by Guarantee)*

**Published:** 1921-02-05

**Authors:** 


### THE COLLEGE OF NURSING

*(Limited by Guarantee).*

### WHAT THE LOCAL CENTRES ARE DOING.

### LONDON CENTRE.

(Secretary, Miss Bompas, 7 Henrietta Street, W. 1.)

Important Notice.

On Monday, February 7, at 8 p.m., at 56 Queen Anne Street, the College of Ambulance, a Lecture by Sir Robert Armstrong-Jones, M.D., on "The Relation between Mental Nursing and Hospital Nursing" will be given to the members of the London Centre. All members are invited to come and to bring their friends. (See remarks on p. 435.)

The Year's Progress.

A general meeting of the London Centre was held on January 25 at Guy's Hospital. Miss Biggar, the Hon. Secretary, read the minutes of the meeting of November 30, in the course of which it was shown that in 1920, although 4,000 more voting papers were sent out than in 1919, 1,000 fewer were returned. She read a letter from Dame Sidney Browne, the President of the Centre, apologising for her absence. Dame Sidney continued that she would be seventy-two next birthday, and thought it was reasonable, as she could not always attend the meetings, that she should resign the presidency. Miss Hughes, however, put it to the meeting that her resignation should not be accepted, and this resolution was unanimously and enthusiastically carried. Miss Biggar then proceeded to give an account of a meeting held on January 20 to consider reports from Local Centres, when fifteen such reports were before them. Three points were emphasised: (1) That the well-represented Centres were perfectly willing to help those needing representation; (2) that the official candidate of a certain Centre might give an election address which should be sent to other Centres; (3) that on the voting paper publicity should be given to the fact that a certain candidate was supported by such-and-such a Centre.

Miss Copeman, the Hon. Treasurer of the Centre, then presented her report, which shows the receipts for 1920, including a balance of £324 brought forward, to be £684, and the expenditure £344. £250 was invested in War Loan, which brings the total so invested to £2,650, a figure which was cheered by the meeting. The balance carried forward is £90. In the absence through indisposition of Miss Bompas, Miss Cowlin presented the Secretary's report, which shows an increase in the membership of 315 since last April, making the total membership 853. She pointed out that there was a disappointing lack of interest in the monthly lectures which are given, and at one, by one of the highest authorities on his subject, only three members attended. Miss Cowlin moved a resolution of thanks to the past and present members of the Council for their work on behalf of the College and the nursing profession, which was passed unanimously.

Miss Rundle's Announcements.

Before the conclusion of the meeting Miss Rundle made the announcement of Lord and Lady Cowdray's munificent gift of £100,000, which we dealt with in our last issue (p. 414), and in the course of her remarks she paid an eloquent and earnest tribute to the great work done by Sir Cooper Perry for the College and nurses, a work little realised perhaps by the body of nurses, because it is done in the quietest and most unobtrusive manner. She further cheered the meeting by announcing that in the new Club-house there would be a library, and that a gift of £500 had been promised for the purchase of books from the Carnegie United Kingdom Trust Fund. After the jam, she presented the meeting with the powder by pointing out that of the 19,500 referendum papers which were sent out (at a cost of £120) only 4,000 replies have been received. But it is refreshing that over 5,000 founder-members have promised annual subscriptions of from one guinea to half-a-crown, that 300 have sent donations, and that 171 of the 5,000 have compounded their annual subscriptions by sending £5 each. The Council requires 5,000 more annual subscriptions before the end of the financial year (March 31), and confidently hopes to receive them. Miss Rundle threatened some home truths, but repented, and the meeting closed on a confident note with hearty votes of thanks to the Governors of Guy's Hospital for the use of the room, and to Miss Hughes for presiding.

[We reported in last week's issue (p. vi) the proceedings at this meeting in relation to nominations for the coming elections.]

### BIRMINGHAM THREE COUNTIES CENTRE.

(Hon. Secretary, Mrs. M. Glegg, 84 Hagley Road.)

Tuesday, February 8, 5.30 p.m., in the Lecture Theatre of the General Hospital, Birmingham (by kind permission of the Governors), address by W. Binwell, Esq., on "The Organisation of the Middle and Professional Classes," with special reference to the Nursing Services. Open to members and all trained nurses. Admission free.

Business and Pleasure.

On Saturday, January 29, a business meeting was held in the Lecture Theatre of the General Hospital, Birmingham (by kind permission of the Governors). After the reports of the Hon. Secretary and the Hon. Treasurer had been proposed and adopted, Miss Musson, R.R.C., the Local Representative, gave a practical and interesting account of some of the work accomplished by the College Council during the past year, and urged upon the members present the need of a recruiting campaign to enlarge the membership of the Centre. Subsequently tea was served in the Board Room, and Nurses Garner, Brown, Chivers, and Bullons, of the General Hospital, sang and played to the assembled members.

### BRIGHTON AND HOVE CENTRE.

(Hon. Secretary, Miss Latham, Royal Sussex County Hospital.)

A Saturday Dance.

In aid of the College Club at Brighton a Saturday dance will be given at the Royal Pavilion, Brighton, on Saturday, February 5, from 8 p.m. to midnight. Tickets, 10s. each, including refreshments, can be obtained from Messrs. Murdoch & Co., 164 Western Road, Brighton.

### A CARDIFF CENTRE CONSTITUTED.

After listening to the powerful advocacy of Sir Arthur Stanley, a public meeting, held at the Cardiff City Hall on Friday, January 28, decided that it was desirable to establish a Centre of the College of Nursing in the Welsh metropolis. There appeared to be rather more than an open mind on the question when the meeting opened, and the Lord Mayor, who presided, cautiously observed that if, after hearing Sir Arthur, he was convinced of the need of a College of Nursing Centre, he would give his support to the project. At the end of Sir Arthur's address, when Alderman Dr. James Robinson proposed the formation of a Local Centre, the Lord Mayor intimated that he was quite satisfied with what he had heard as to the objects of the College, and would accord it his utmost support. Sir William Diamond expressed the hope that the project would not tend to make nurses selfish, and so destroy the splendid spirit which dominated the profession. Miss Sherriff MacGregor, Organising Secretary of the College, was present, and emphasised the fact that if the College is truly to represent the nurses it must have the active interest of all of them. Afterwards the Cardiff Centre was formally constituted. Sir William Jas. Thomas was elected to be President, and Miss Hitch, Sister-Tutor of King Edward VII. Hospital, to be Hon. Secretary.

### NORFOLK AND NORWICH CENTRE.

(Hon. Secretary, Miss V. A. Rutter, 43 Newmarket Road.)

Forthcoming Lectures.

Thursday, February 3, 8 p.m., at Norfolk and Norwich Hospital, "A Constantinopolitan Excursion," by H. Cooper Pattin, Esq., M.D.

Thursday, February 17, 8 p.m., at Jenny Lind Hospital, "The Commoner Diseases of the Ear," by N. S. Carruthers, Esq., F.R.C.S.

Thursday, March 3.—Lecture to be notified later.

Thursday, March 17, 8 p.m., at the Eye Infirmary, "The Eye and its Diseases in Relation to the Nursing Profession," by G. Maxted, Esq.

Thursday, April 7, 3 p.m., a visit to the Castle Museum (by kind permission), conducted by F. Leney, Esq., Curator.

Thursday, April 21, 8 p.m., at the Norfolk and Norwich Hospital (by kind permission), "Travels," by Sir Eustace Gurney, with lantern slides.

Members are admitted free, but local membership cards must be shown at each lecture. Non-members will be welcome on payment of 1s. each.

### The College of Nursing.

**(Limited by Guarantee.)**

(*Continued from page* vi.)

### EAST LANCASHIRE CENTRE.

(Hon. Secretary, Miss Earl, Ancoats Hospital, Manchester.)

Mrs. Bedford Fenwick has accepted this Centre's invitation to give an address at a meeting to be held on an early date. Particulars will be announced later.

### SHEFFIELD CENTRE.

(Hon. Secretary, Mrs. J. Barnes, 34 Wilkinson Street.)

Sheffield is working hard for its own College Club, and recently a ball was given at the Cutlers' Hall in aid of the funds. Mrs. Watson, Hon. Treasurer Local Centre, was the organiser, and she is to be heartily congratulated on her efforts. The medical profession was largely represented by some of its leading members and their ladies; the matrons from all the local hospitals were present; and the civic life was represented by the presence of the Master and Mistress Cutler (Mr. and Mrs. W. Clarke). Though uniform was worn by some nurses, many sisters and nurses were in mufti. Dancing continued until the early hours of the morning. Mr. Callum's band was responsible for the delightful music.

### YORKSHIRE CENTRE, LEEDS.

(Hon. Secretary, Miss M. V. Lindall, Hospital for Women and Children.)

Friday, February 11, 7 p.m., in the Clinical Theatre of the Leeds General Infirmary, members' meeting. Address by Miss E. Sheriff Macgregor, R.R.C. (Organising Secretary, the College of Nursing), on "Roumania and Russia in War Time." A large attendance is hoped for, as the question of nominating a candidate for the coming Council election will be brought up at this meeting.

### SWANSEA AND SOUTH WALES CENTRE.

(Hon. Secretary, Mrs. F. W. Jenner, Glynn Vivian Home of Rest for the Blind, Caswell Hill.)

Last Week's Meetings.

On Friday, January 28, a lecture was given in the Y.M.C.A., Swansea, by Dr. Mary Scharlieb, C.B.E., on "Venereal Disease as it Affects Women and Children." The lecturer dealt with her subject in a straightforward and outspoken manner, which she combined with tact and delicacy, and put forward a plea for the inauguration of Centres of the "National Society for the Prevention and Cure of Venereal Disease" throughout the country, especially in large industrial and seaport towns. Dr. Scharlieb also pointed out the obligation which the new dignity of full citizenship and suffrage entails on women to use their influence more fully in all matters of public health.

On the following afternoon a public meeting was held in the Y.M.C.A., under the chairmanship of Mr. Stanley Cook, when Sir Arthur Stanley, G.B.E., C.B., Chairman of the Council of the College of Nursing, spoke on "The College of Nursing and the work of its Local Centres."

An interesting discussion followed, at the conclusion of which a hearty vote of thanks to Sir Arthur Stanley was proposed by the Rev. H. C. Manderand, seconded by Dr. Nelson Jones, in which a tribute was paid to the fair and straightforward manner in which the proceedings had been conducted.

